# Prognostic biomarkers in COVID-19 infection: value of anemia, neutrophil-to-lymphocyte ratio, platelet-to-lymphocyte ratio, and D-dimer

**DOI:** 10.1186/s43168-021-00075-w

**Published:** 2021-05-21

**Authors:** Maiada K. Hashem, Eman M. Khedr, Enas Daef, Aliae Mohamed-Hussein, Ehab F. Mostafa, Sahar M. Hassany, Hanan Galal, Shimaa Abbas Hassan, Islam Galal, Mariam Taher Amin, Hebatallah M. Hassan

**Affiliations:** 1 Department of Chest Diseases, Faculty of Medicine, Assiut University, Assiut, Egypt; 2 Department of Neurology and Psychiatry, Assiut University, Assiut, Egypt; 3 Department of Medical Microbiology and Immunology, Faculty of Medicine, Assiut University, Assiut, Egypt; 4 Department of Tropical Medicine and Gastroenterology, Alrajhi University Hospital, Assiut University, Assiut, Egypt; 5 Clinical Pathology Labs, General Chest Hospital, Assiut, Egypt; 6 Department of Anesthesia and Intensive Care, Assiut University, Assiut, Egypt; 7 Department of Chest, Faculty of Medicine, Aswan University, Aswan, Egypt; 8 Department of Public Health and Community Medicine, Assiut University, Assiut, Egypt

**Keywords:** COVID-19, SARS-CoV-2, Severity, ICU admission, Neutrophil-to-lymphocyte ratio, NLR, Platelets-to-lymphocyte ratio, PLR, Anemia, D-dimer

## Abstract

**Background:**

Being highly infectious disease, COVID-19 exhausts most of efficient healthcare systems worldwide. Simple and rapid risk stratification methods are mandatory to recognize severe patients. This study aims to highlight the simple available laboratory biomarkers of good predictive value for COVID-19 severity.

**Results:**

Three hundred fifty-one COVID-19 positive patients admitted to two University Hospitals between the 1st of June and the 31st of July 2020 were retrospectively collected and classified to severe and non-severe COVID-19 patients according to need for ICU admission. All basic laboratory biomarkers at time of admission were recorded. Of included patients, 145 (41.3%) needed ICU admission. Anemia, leukocytosis, lymphopenia, NLR, and PLR together with liver enzymes, INR, ferritin, CRP, and D-dimer were significantly higher in patients needed ICU admission (*p* < 0.001). However, by applying multivariate logistic regression, only anemia, high NLR, high PLR, and high D-dimer levels showed significant risk for ICU admission with OR equal 3.6 (95% CI 1.8–7.0), 9.0 (95% CI 3.6–22.6), 3.0 (95% CI 1.3–7.1), and 2.5 (95% CI 1.3–4.7), respectively.

**Conclusion:**

Anemia, increased neutrophil-to-lymphocyte ratio (> 8), platelet-to-lymphocyte ratio (> 192), and D-dimer level (> 0.9 mg\L) at time of admission could be simple available predictors for severe COVID-19 infection requiring ICU admission.

## Background

Since being declared as a pandemic by the WHO on March 2020, COVID-19 infection posed great threat to human health consuming most of resources of efficient healthcare systems. Numerous hospitals, globally, are presently suffering a lack of ICU beds for critically ill COVID-19 patients. A hazard stratification established on clinical, radiological, and laboratory considerations appears essential to better categorize those patients who may requisite hospital or ICU admittance. Numerous laboratory biomarkers are used initially for COVID-19 infection prediction or diagnosis; however, their accuracy to assess infection severity and prognosis as well as the levels at which they are alarming results are still to be evaluated [1].

Lymphopenia, leukocytes, and high neutrophil count are simple initial parameters proposed to directly discriminate between severe and non-severe COVID-19 patients [2, 3]. T cells play a critical role in antiviral immunity, though; the elements which might cause the decline in count, and the state of the activation of T cells in COVID-19 cases remain largely unclear [4]. Increased prothrombin time and D-dimer values may also be indicators of a worse prognosis [5, 6] which is explained by dysregulated coagulopathy in severe COVID-19 patients. Inflammation-related proteins seem also to provide valuable prognostic data. Elevated procalcitonin, C-reactive protein (CRP) levels, and serum ferritin distinguish between mild and severe COVID-19 cases [2, 7]. Other inflammatory cytokines such as Interleukin-2R (IL-2R) and Interleukin-6 (IL-6) and biochemical factors including liver enzymes, kidney function tests, and lactic dehydrogenase (LDH) may also be markedly altered in severe COVID-19 patients [8–10].

This study aims to highlight the simple available laboratory biomarkers of good predictive value for COVID-19 severity and find the accurate cut-off points for those markers.

## Methods

This was a retrospective observational cohort study conducted on COVID-19 patients admitted to Assiut and Aswan University Hospitals as tertiary hospitals in the period between the 1st of June and the 31st of July 2020. All participants were diagnosed with COVID-19 according to the WHO and Egyptian Ministry of Health and Population (MOHP) definitions [11, 12]. Diagnosis of the cases was confirmed using RT-PCR for detection of the viral RNA by TaqMan™ 2019-nCoV Control Kit v1 (Cat. No. A47532) supplied by QIAGEN, Germany on the Applied Biosystem 7500 Fast RT PCR System, USA. It was a total coverage sample included all patients admitted during the study period in study hospitals.

The inclusion criteria included all adult hospitalized COVID-19 patients of both genders and all disease severity levels. After reviewing of records, any patient younger than 18 years or with missed laboratory data or primary outcome status was excluded from the study.

### Data collection

Clinical records and laboratory data were reviewed by the investigators in each study site and the following data were extracted for analysis:

1. *Demographic and clinical data*: age, gender, presenting symptoms, comorbidities, and outcomes.
2. *Laboratory investigations:* complete blood count (by Pentra 80 Horiba blood counter), neutrophil-to-lymphocyte ratio (NLR) and platelet-to-lymphocyte ratio (PLR) were then calculated by dividing the absolute count of neutrophils and platelets by the absolute count of lymphocytes respectively, D-dimer (on sysmex 1500) and serum ferritin (by Advia centaur). In addition, C-reactive protein “CRP”, serum urea, creatinine, and liver enzymes (alanine aminotransferase “ALT” and aspartate aminotransferase “AST”).
3. *Chest CT findings.*
4. *Clinical outcomes*: the primary outcome of the study was intensive care unit admission. Patients admitted to ICU if they had one or more of the following [12]:
  Respiratory rate more than 30 cycles/min.
  Arterial partial pressure of oxygen (PaO2)/fraction of inspired oxygen (FiO2) less than 300 mmHg or respiratory failure requires mechanical ventilation.
  Presence of shock or other organ failure requires monitoring or treatment in ICU.

Based on this outcome, patients were divided into:

- Group I: COVID-19 patients who were not admitted to ICU
- Group II: COVID-19 patients who were admitted to ICU

### Ethical approval

The study protocol was approved by the ethical committee of the Faculty of Medicine, Assiut University (IRB no. 17300434). Patients’ records were retrospectively reviewed after IRB waiver of consent. Patients identifying information were concealed and each patient assigned for a code to insure privacy and confidentiality of the data. It was conducted in accordance with the provisions of the Declaration of Helsinki.

### Statistical analysis

All statistical analyses were performed using IBM SPSS Statistics version 20 (SPSS Inc., Chicago, IL, USA). Categorical data were presented as numbers and percentages, while chi-square tests were used for comparisons between groups. Continuous data were reported as means ± SD and tested for normality using the Shapiro-Wilk test. As all laboratory data were not normally distributed, the Mann-Whitney test was used for comparison between groups. For optimal cut-off points for laboratory data, we used ROC analysis. Not all optimal cut-off points were consistent with laboratory reference levels. For laboratory parameters with optimal cut-off points different from the reference levels, we used the new cut-off points after ROC analysis with reporting of AUC, sensitivity, and specificity for the new values. Logistic regression models were fit for ICU admission status as dependent variables, and all significant demographic and laboratory variables were included as independent variables. Laboratory variables were converted to dichotomous variables based on optimal cut-off values. First univariate logistic regression was conducted, and then all the significant values were included in the final model of multivariate logistic model. In all statistical tests, *p* value < 0.05 was considered statistically significant.

## Results

**Fig. 1:**
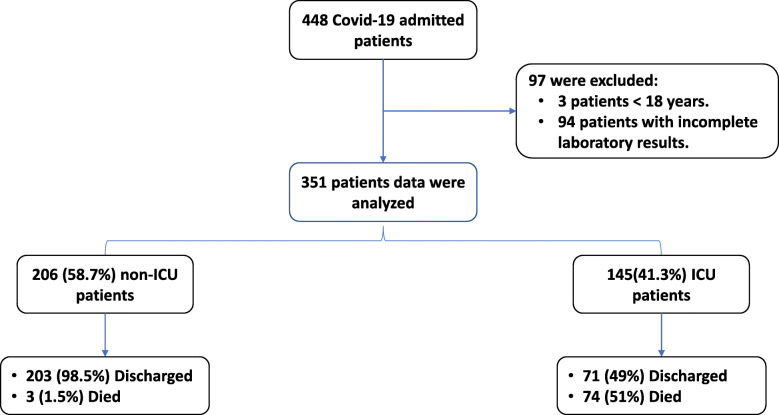
Flowchart of study participants

Screening of 448 patients’ records was conducted and 3 patients < 18 years old as well as 94 patients with incomplete data were excluded. Analysis of 351 cases was then performed, and 145 (41.3%) needed ICU admission (Fig. 1).

The mean age of patients admitted to ICU was significantly higher than that of those who did not need ICU admission (57.4 ± 14.0 vs 45.1 ± 17.1, *p* < 0.001) besides 58.6% of them were males. As regards the presenting symptoms, fever, and lower respiratory tract (LRT) symptoms especially dry cough were the most predominant symptoms in both groups but significantly higher in patients needed ICU admission. The presence of comorbid diseases was higher in the same group as well (*p* < 0.001) (Table 1).

**Table 1:** Demographic and clinical characteristics at admission of COVID-19 patients included in the study (*n* = 351)

	Non-ICU patients (***n*** = 206)	Admitted to ICU (***n*** = 145)	***p*** value*
**Age (years)**			
**< 55,** ***n*** **(%)**	135 (65.5%)	52 (35.9%)	< 0.001**^**
**≥ 55,** ***n*** **(%)**	71 (34.5%)	93 (64.1%)	
**Mean ± SD**	45.1 ± 17.1	57.4 ± 14.0	< 0.001**^**
**Gender,** ***n*** **(%)**			
**Male**	98 (47.6%)	85 (58.6%)	0.041 **^**
**Female**	108 (52.4%)	60 (41.4%)	
**Presenting symptoms,** ***n*** **(%)**			
**Fever**	138 (67%)	124 (85.5%)	< 0.001 **^**
**Constitutional symptoms**	57 (27.7%)	65 (44.8%)	0.001 **^**
**Cough**	129 (62.6%)	117 (80.7%)	< 0.001 **^**
**Dyspnea**	70 (34%)	103 (71%)	< 0.001 **^**
**GIT symptoms** ^***a***^	47 (22.8%)	21 (14.5%)	0.071
**Presence of comorbidity,** ***n*** **(%)**	94 (45.6%)	104 (71.7%)	< 0.001 **^**

*GIT* gastrointestinal

*Student’s *t* test and chi-square test were used

**^**Significant *p* valu*e*

^**a**^Nausea, vomiting, abdominal pain, and diarrhea

Anemia, leukocytosis, and lymphopenia were significantly present in patients needed ICU admission (*p* < 0.001); however, no significant statistical difference recognized in the platelet count between both groups. Both NLR and PLR were considerably higher in ICU patients’ group when using different reference ranges in literatures and by using the optimum cut-off points in this study which were > 8 and > 192, respectively, (*p* < 0.001). Significant lower albumin level, higher total bilirubin levels, liver enzymes, INR, blood urea nitrogen, and serum creatinine levels were all found in COVID-19 patients admitted to ICU. Furthermore, those patients had higher levels of CRP, serum ferritin, and D-dimer than ward patients (*p* < 0.001) (Table 2).

**Table 2:** Laboratory data of COVID-19 patients included in the study (*n* = 351)

	Non-ICU patients (***n*** = 206)	Admitted to ICU (***n*** = 145)	***p*** value*
**CBC**			
**Hemoglobin (g/dl)**	12.7 ± 1.7	11.8 ± 2.0	< 0.001 **^**
***Anemic (HB < 12)***	59 (28.6%)	82 (56.6%)	< 0.001 **^**
**WBCs (10**^**3**^**/ul)**	7.9 ± 4.5	10.6 ± 6.1	< 0.001 **^**
***Low (< 4)***	31 (15.1%)	11 (7.6%)	< 0.001 **^**
***High (> 10)***	49 (23.8%)	72 (49.7%)	
**Neutrophil (10**^**3**^**/ul)**	5.7 ± 4.5	8.7 ± 5.5	< 0.001 **^**
***High (> 7)***	67 (32.5%)	92 (63.4%)	< 0.001 **^**
**Lymphocytes (%)**	24.7 ± 14.3	13.0 ± 12.7	< 0.001 **^**
***Low (< 20)***	99 (48.1%)	115 (79.3%)	< 0.001 **^**
**Lymphocytes (10**^**3**^**/ul)**	1.6 ± 0.9	1.0 ± 0.8	< 0.001 **^**
***Low (< 1.5)***	119 (57.8%)	116 (80%)	< 0.001 **^**
**Platelets (10**^**3**^**/ul)**	273.3 ± 131.5	279.5 ± 137.4	0.672
***High (> 450)***	20 (9.7%)	14 (9.7%)	0.987
**NLR**	4.7 ± 5.0	15.5 ± 23.2	< 0.001 **^**
***High (> 8)***	29 (14.1%)	89 (61.4%)	< 0.001 **^**
**PLR**	231.8 ± 207.4	417.2 ± 342.7	< 0.001 **^**
***High (> 192)***	83 (40.3%)	108 (74.5%)	< 0.001 **^**
**INR**	1.04 ± 0.12	1.13 ± 0.24	< 0.001 **^**
***High (≥ 1.1)***	31 (15%)	51 (35.2%)	< 0.001 **^**
**Liver function tests**			
**Albumin (g/L)**	33.6 ± 6.2	30.6 ± 6.9	< 0.001 **^**
***Low (< 34)***	118 (57.3%)	99 (68.3%)	0.037
**Total bilirubin (umol/L)**	7.6 ± 6.5	11.7 ± 11.5	< 0.001 **^**
***High (> 21)***	7 (3.4%)	10 (6.9%)	0.133
**AST (U/L)**	42.8 ± 69.5	67.1 ± 101.9	< 0.001 **^**
***High (> 34)***	90 (43.7%)	85 (58.6%)	0.006
**ALT (U/L)**	43.8 ± 65.3	73.7 ± 149.7	0.001 **^**
***High (> 45)***	58 (28.2%)	67 (46.2%)	0.001 **^**
**Kidney function tests**			
**Serum creatinine (umol/L)**	100.0 ± 70.8	141.3 ± 142.9	0.022 **^**
***High (> 97)***	82 (39.8%)	68 (46.9%)	0.186
**BUN (mmol/L)**	13.8 ± 15.8	18.7 ± 19.2	< 0.001 **^**
***High (> 8.2)***	90 (43.7%)	105 (72.4%)	< 0.001 **^**
**Ferritin (ng/ml)**	491.4 ± 608.9	1161.5 ± 1321.1	< 0.001 **^**
***High (> 440)***	69 (33.5%)	103 (71%)	< 0.001 **^**
**D-dimer (mg/L)**	1.3 ± 2.3	4.0 ± 6.8	< 0.001 **^**
***High (> 0.9)***	66 (32%)	106 (73.1%)	< 0.001 **^**
**CRP (mg/dl)**	37.9 ± 49.5	80.7 ± 76.1	< 0.001 **^**
***High (> 23)***	97 (47.1%)	111 (76.6%)	< 0.001 **^**

*CBC* complete blood picture, *WBCs* white blood cells, *NLR* neutrophil lymphocytic ratio, *PLR* platelets lymphocytic ratio, *INR* international normalization ratio, *AST* aspartate aminotransferase, *ALT* alanine aminotransferase, *BUN* blood urea nitrogen, *CRP* C-reactive protein

*Mann-Whitney and chi-square test were used.

**^**Significant *p* value

**Fig. 2:**
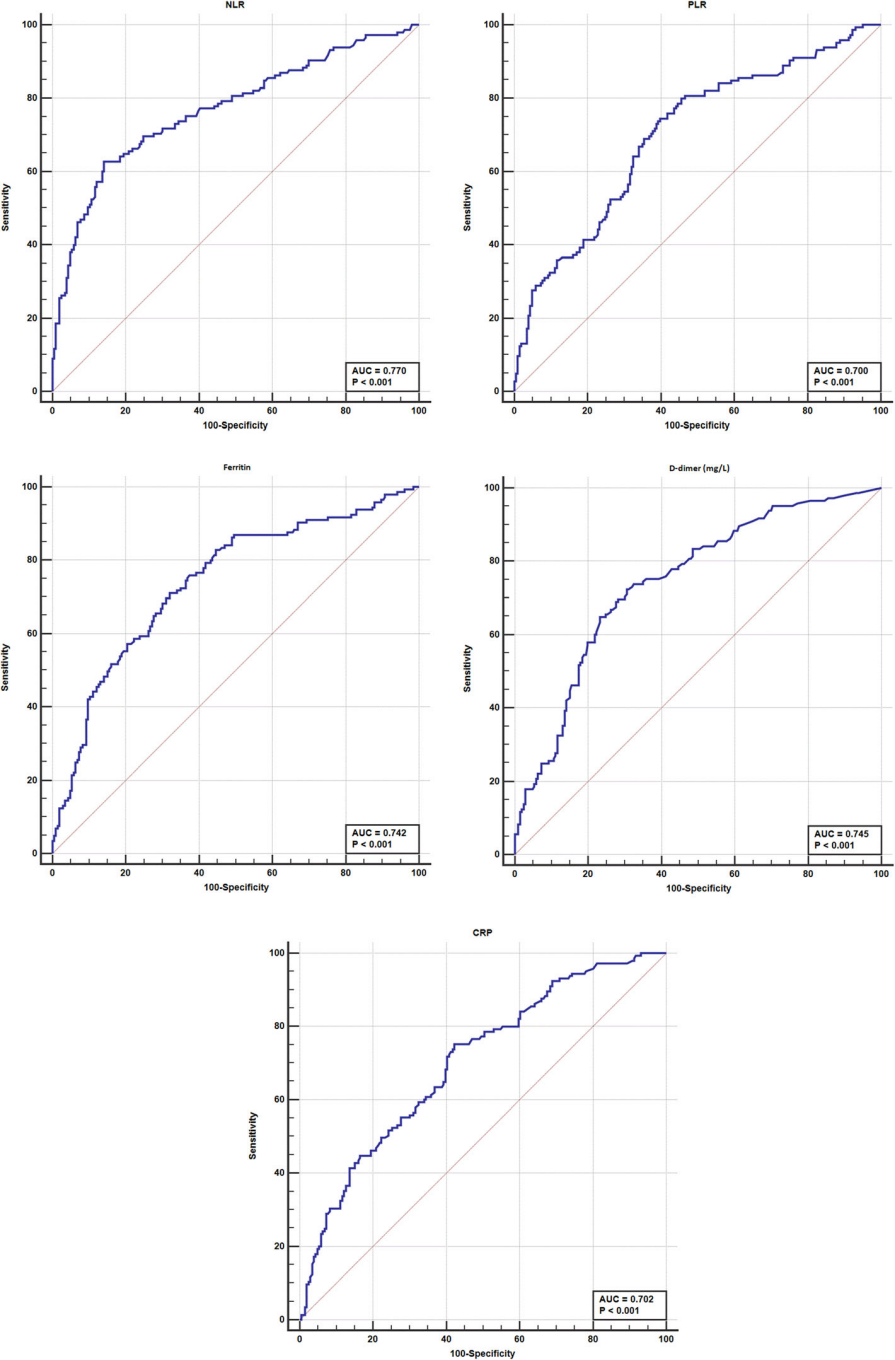
ROC curves for selected laboratory parameters (NLR, PLR, Ferritin, D-dimer, CRP) in ICU and non-ICU admitted COVID-19 patients (*n* = 351)

The proposed optimum cut-off point for NLR that could predict COVID-19 severity and ICU admission was > 8 showed sensitivity = 60.7%, specificity = 85.9%, and AUC = 0.770. A level of PLR > 192 showed sensitivity = 74.5%, specificity = 60.2%, and AUC = 0.700. A level > 440 ng/ml serum ferritin showed the best predictive accuracy for ICU admission with sensitivity = 71%, specificity = 67.5%, and AUC = 0.742. D-dimer level above 0.9 mg/l had the best value to predict ICU admission for COVID-19 patients with sensitivity = 72.4%, specificity = 68.9%, and AUC = 0.745. Regarding CRP, the cut-off point was > 23 with sensitivity = 75.2%, specificity = 53.9%, and AUC = 0.702 (Fig. 2),

In univariate logistic regression model, the older ages had higher risk of ICU admission than lower age groups as OR for age above 50 years was 3.4 (95% CI 2.2–5.3). Male gender also carried higher risk as OR was 1.6 (95% CI 1.0–2.4). Among different presenting symptoms, fever, cough, and dyspnea were associated with substantial increased risk for ICU admission as well as patients with comorbid conditions. For different laboratory biomarkers, presence of anemia, high neutrophile count, low lymphocyte count, high NLR and PLR, impaired liver and kidney functions, high serum ferritin, D-dimer, and CRP carried increased risk for ICU admission. However, after adjustment of significant factors in multivariate logistic regression, only anemia, high NLR, high PLR, and high D-dimer levels showed higher risk for ICU admission with OR equal 3.6 (95% CI 1.8–7.0), 9.0 (95% CI 3.6–22.6), 3.0 (95% CI 1.3–7.1), and 2.5 (95% CI 1.3–4.7), respectively (Table 3).

**Table 3:** Logistic regression for predictors of ICU admission

Factors	Univariate analysis		Multivariate analysis	
	OR (95% CI)	***p*** value	OR (95% CI)	***p*** value
**Age (≥ 55 years)**	3.4 (2.2–5.3)	< 0.001**^**	0.87 (0.420–1.8)	0.709
**Sex (Ref**. = female**)**				
Male	1.6 (1.0–2.4)	0.042**^**	1.8 (0.94–3.5)	0.075
**Fever**	2.9 (1.7–5.0)	< 0.001**^**	1.1 (0.49–2.6)	0.774
**Constitutional symptoms**	2.1 (1.4–3.3)	0.001	1.3 (0.69–2.6)	0.390
**Cough**	2.5 (1.5–4.1)	< 0.001**^**	1.0 (0.48–2.2)	0.971
**Dyspnea**	4.8 (3.0–7.6)	< 0.001**^**	2.3 (1.1–5.0)	0.032**^**
**Presence of comorbidity**	3.0 (1.9–4.8)	0.001**^**	3.0 (1.4–6.3)	0.004**^**
**Anemic (HB < 12 g/dl)**	3.2 (2.1–5.1)	< 0.001**^**	3.4 (1.7–6.5)	< 0.001 **^**
WBCs (Ref. = 4–10*10^**3**^**/ul)**				
Low (< 4)	0.72 (0.34–1.5)	0.394	1.4 (0.45–4.6)	0.541
High (> 10)	3.0 (1.9–4.8)	< 0.001	1.03 (0.48–2.2)	0.934
High neutrophil (> 7*10^**3**^**/ul)**	3.6 (2.3–5.6)	< 0.001**^**	1.2 (0.40–3.6)	0.760
**Low lymphocyte % (< 20)**	4.1 (2.5–6.7)	< 0.001**^**	0.63 (0.26–1.5)	0.303
Low absolute lymphocytes (< 1.5*10^**3**^**/ul)**	2.9 (1.8–4.8)	< 0.001**^**	0.61 (0.24–1.6)	0.327
**High NLR (> 8)**	9.7 (5.8–16.2)	< 0.001**^**	7.2 (2.9–17.7)	< 0.001 **^**
**High PLR (> 192)**	4.3 (2.7–6.9)	< 0.001**^**	3.2 (1.4–7.7)	0.008 **^**
**INR (≥ 1.1)**	3.1 (1.8–5.1)	< 0.001**^**	1.8 (0.86–3.9)	0.119
**Low albumin (< 34 g/L)**	1.6 (1.0–2.5)	0.037**^**	0.922 (0.49–1.8)	0.806
**High AST (> 34 U/L)**	1.8 (1.2–2.8)	0.006**^**	1.3 (0.64–2.5)	0.496
**High ALT (> 45 U/L)**	2.2 (1.4–3.4)	0.001**^**	1.9 (0.93–4.0)	0.079
**High BUN (> 8.2 mmol/L)**	3.4 (2.1–5.3)	< 0.001**^**	1.6 (0.86–3.1)	0.135
**High ferritin (> 440 ng/ml)**	4.9 (3.1–7.7)	< 0.001**^**	1.5 (0.77–3.0)	0.226
**High D-dimer (> 0.9 mg/L)**	5.8 (3.6–9.2)	< 0.001**^**	2.3 (1.2–4.3)	0.011 **^**
**High CRP (> 23 mg/dl)**	3.7 (2.3–5.9)	< 0.001**^**	1.3 (0.62–2.)	0.519

*HB* hemoglobin, *WBCs* white blood cells, *NLR* neutrophil lymphocytic ratio, *PLR* platelets lymphocytic ratio, *INR* international normalization ratio, *AST* aspartate aminotransferase, *ALT* alanine aminotransferase, *BUN* blood urea nitrogen, *CRP* C-reactive protein

**^**Significant *p* value

## Discussion

The number of patients acquiring COVID-19 infection are dramatically increasing globally affecting the efficiency of healthcare systems specially ICU bed availability. Therefore, early detection of severe cases is mandatory for rapid triaging of patients. While the clinical presentation, associated comorbidities, extent of radiological infiltration, and the blood oxygen saturation of COVID-19 patients may indicate the need for their admittance to ICUs, several laboratory parameters may facilitate the assessment of disease severity.

This study included 351 patients, of them 145 (41.3%) admitted to ICU. Patients needed ICU admission were significantly older age, predominantly males with significant higher frequency of fever, dyspnea, and cough as well as concomitant comorbid conditions. They also had significant anemia, leukocytosis, lymphopenia, and higher NLR and PLR (*p* < 0.001). Impaired INR, liver functions, and kidney functions were also more significant in patients admitted to ICU. Serum ferritin, CRP, and D-dimer were significantly higher as well (*p* < 0.001). However, by applying multivariate regression analysis, only high NLR (> 8), PLR (> 192), and D-dimer (> 0.9 mg/l) were the significant risk factors that associated with severe COVID-19 infection needed ICU admission.

Complete blood count is one of the essential widely available investigations for COVID-19 infection diagnosis and severity assessment. Anemia is suggested to be associated with an increased risk of severe COVID-19 infection [13]. In this study, anemia is defined when the hemoglobin (Hb) levels were below 12 g/L. In literatures, the cut-off point of Hb level that define anemia associated with severe COVID-19 infection ranges from 11 to 13 g/L [14–16]. Anemia may increase risk for severe COVID-19 infection by the following mechanisms: (A) low hemoglobin level is associated with decreased oxygen delivery to organs and tissues which may aggravate hypoxemia especially to respiratory system thus play an important role in the development of multi-organ failure [17]. (B) SARS-CoV-2 can interact with ACE2, CD147, and CD26 receptors on the erythrocyte. This interaction between the virus and the hemoglobin will cause the virus to strike the heme on the beta-1 chain of hemoglobin and causing hemolysis [18]. (C) The hepcidin-mimetic action of SARS-CoV-26 may induce ferroptosis—hepcidin is down regulated by low serum iron, resulting in high oxidative stress and lipoperoxidation that can precipitate the inflammatory/immune over-response causing severe disease [18]. On the other hand, some studies found no link between anemia and disease severity or poor prognosis [19, 20]. In current study, anemia not only was significantly associated with severe COVID-19 patients but also it was recognized to be a risk factor for severe disease by multivariate regression analysis.

Leukocytosis and lymphopenia are also suggested to be hazard factors for severe COVID-19 infection and poor outcome [21, 22]. A recent meta-analysis of 10 studies conducted that lower lymphocyte and higher leukocyte counts were allied with severe infection [22]. Lymphopenia was formerly used as prognostic biomarker in other infectious diseases such as influenza. Association between lymphopenia and severe disease may be the consequence from direct infection of the lymphocyte, lymphatic tissue destruction, lymphocyte apoptosis due to inflammation, or some metabolic abnormalities for instance lactic acidosis causing lymphocytes inhibition [23]. Despite being associated with severe COVID-19 infection in this study, neither leukocytosis nor lymphopenia were a significant risk factor by multivariate regression analysis.

On the other hand, increased neutrophil-to-lymphocyte ratio at admission was found to be an independent risk factor for severe disease and mortality in COVID-19 patients [20, 24–26]. Neutrophil-to-lymphocyte ratio is stress and immune parameter. For COVID-19, the increased neutrophils indicate the degree of the inflammatory response, and the decreased lymphocytes indicate the degree of immune imbalance. These associations are amplified by the concept of NLR [27]. The normal values of NLR in adults are in the range between 1.0 and 2.3. The cut-off value of NLR that could predict poor COVID-19 infection varies widely among literatures. It ranges between 3.13 and 9.38 [24, 27]. In the current study, the optimum cut-off point that could predict severe COVID-19 infection was > 8. A large cohort study on similar population found a statistically significant strong association of in-hospital mortality with neutrophil-lymphocyte ratio > 3.1 [28]. The lack of a single universal definition for severe COVID-19 infection and the variable outcome measures used in different studies could explain this wide range and variances. However, there is agreement about the value of elevated baseline NLR in predicting severe COVID infection.

Platelet-to-lymphocyte ratio is another simple cost-effective but less commonly used method that is calculated from CBC and could be of a value in predicting COVID-19 severity. Two recent meta-analysis evaluating PLR value in COVID-19 infection showed that cases with severe COVID-19 had higher admission levels of PLR [29, 30]. Moreover, the number and the dynamic fluctuations of the platelets during the management may provide an idea on the prognosis and severity of the illness. The cytokine storms in the affected patient were associated with prominently higher platelet count and longer mean hospitalization days. The PLR of the patient reflects the degree of cytokine storm, which might deliver a novel sign in the monitoring of patients with COVID-19 [31].

Regarding inflammatory biomarkers associated with the COVID-19 infection, many meta-analysis studies observed higher concentrations of C-reactive protein (CRP) among patients with severe COVID-19 infection [32–34]. In the current study, higher levels of CRP were found in patients with severe COVID-19 infections admitted to ICU. However, it was not found to be a predictor for severity by applying multivariate regression analysis even with chosen relatively high and sensitive—but not specific—cut-off point.

Coronavirus disease 2019 can affect coagulation and hemostasis by different mechanisms including both abnormal bleeding risk and thromboembolism. So, all main coagulation biomarkers disturbances were found in COVID-19 cases namely higher serum D-dimer level, longer prothrombin time (PT), and lower platelets counts [35]. Moreover, D-dimer levels correlate with the severity of the disease and are a dependable prognostic indicator for the hospital mortality in the admitted patients with COVID-19. The elevated D-dimer signify a hyperfibrinolysis state and increased inflammatory burden induced in SARS-COV-2 infection [36, 37]. The optimum D-dimer level that could predict worse prognosis varies in literatures between > 1 mg/L [8] to > 2.14 mg/L [36]. In the current study, D-dimer > 0.9 mg\L found to be a sensitive predictor for ICU admission in patients with COVID-19 infection.

The current study had some limitations. The study is based on reviewing medical records of admitted patients and obtained data at admission. Limited number of patients encountered the inclusion criteria. All laboratory values were collected at admission and the follow up results and linear changes in correlation with the clinical condition of the patients were not evaluated. We focused on the simplest and cost-effective investigations, while some other biomarkers such as IL-6 level, LDH, CK-MB, troponin, and procalcitonin were not evaluated.

## Conclusion

Anemia, increased neutrophil-to-lymphocyte ratio (> 8), platelet-to-lymphocyte ratio (> 192) and D-dimer level (> 0.9 mg\L) at time of admission can be simple available predictors for severe COVID-19 infection requiring ICU admission. Future studies are needed to evaluate the linear change of NLR, PLR, and D-dimer with disease progression.

## Data Availability

Data are available.

## Abbreviations

ALT: Alanine aminotransferase
AST: Aspartate aminotransferase
COVID-19: Corona virus disease 2019
CRP: C-reactive protein
Hb: Hemoglobin
ICU: Intensive care unit
IL: Interleukin
LDH: Lactate dehydrogenase
NLR: Neutrophil-to-lymphocyte ratio
PLR: Platelet-to-lymphocyte ratio
SARS-CoV-2: Severe acute respiratory syndrome coronavirus 2
WHO: World Health Organization
